# On the Efficacy of the Tonic and Sedative Plan of Treatment in Cases of Fever and Disturbed Nervous Functions after Delivery

**Published:** 1851-08

**Authors:** T. W. Garlike


### OBSTETRICS.

*On the Efficacy of the Tonic and Sedative Plan of Treatment in Cases of Fever and Disturbed Nervous Functions after Delivery.* By T. W. Garlike, Esq.—In a former paper I endeavored to show that a great proportion of cases, vaguely designated "puerperal fever," are not to be regarded as of sthenic or inflammatory character, dependent upon increased vascular action; but, on the contrary, that they are simply attributable to a morbid sensibility of the nervous system resulting from depression of nervous energy. I am now anxious to illustrate my views on this subject still further; and, as the case I am about to relate is highly corroborative of the views I have advocated, I shall give it in detail.

The case runs thus:—Mrs. ——, aged 43, middle-sized, and of robust appearance, requested my attendance, being in labour, (her first pregnancy,) on the evening of the 4th July last. Her pains were, to all appearance, of a useful "bearing down" character. On making an examination I found the os tincæ had dilated just sufficiently for me to ascertain that it was a vertex presentation. Having represented to Mrs. —— that my services were not required for the present, I left; at the same time I instructed her nurse to call me if any change manifested itself during the night. My patient resided within a short distance of my house. On the morning of the 3rd, Mrs. —— described the pains which had followed up through the previous night as having been of a severe kind, but not forcing. On making an examination I found that the labour had not made much progress; during the day she was tolerably free from pain, and took nourishment; but at night, her pains having returned with more severity, my services were again requested. I stayed through the night; there were occasional active pains, but not sufficiently forcing to be efficacious, At 5 a. m. of the 4th, gave a draught of sulphuric ether and camphor mixture, with 25 drops of laudanum. Visited my patient again at 10 a. m.; the draught had produced some sleep, from which she was refreshed; but, as the day advanced, she manifested symptoms of great prostration; the countenance was anxious and solicitous; skin hot; pulse frequent, small, and irregular; tongue dry, creamy and brown down the centre; tenderness over the hypogastric region, with inability to pass urine.

The symptoms were relieved considerably by the use of the catheter, which was resorted to frequently during the labour.

Ordered saline febrifuge every six hours.

In the evening she appeared much better in all essentials; the temperature of the skin was reduced, tongue moist, bowels acted upon twice, circulation more quiet, and the countenance and conduct more indicative of hopefulness. Although my patient had now been ill two days and nights, during which time she had borne a large proportion of good forcing pains, still, on making an examination, the labour had not advanced much, the os tincæ, though certainly more dilated and yielding, was, nevertheless, rigid, and the lips thick; the head had made some trifling descent, but had not passed the true pelvis, neither did the pains (which were severe) appear to exercise any bearing-down influence upon it. The great impediment appeared to be the rigid, unyielding condition of the osseous structure of the mother, and the highly organized condition, as well as great development of the fœtal cranial bones, which were firmer than I ever met with; there was also a want of room on the antero-posterior diameter of the pelvis.

In carrying out the examination, which was conducted with great care, a symptom presented itself which gave me some concern: I detected crepitation over a limited portion of the mucous membrane in the perineal aspect of the vagina ; when a pain was on, it was clearly made out, and it instantly conveyed to my mind the supposition that it might be, as in other tissues when subjected for a long time either to increased action of the capillaries or the opposite state of these vessels, congestion, the first symptom of sloughing taking place, or the formation of pus.

Looking upon my patient as being in a condition to require watching, I stayed the night, hoping some favorable change might show itself before morning. She stated that she had not felt the movements of the child for some hours.

Ordered a draught, with 30 drops of laudanum.

About midnight, Mrs. —— began to exhibit symptoms of restlessness and delirium, and the circulation began again to take on the characteristics of exhaustion by increasing rapidly its frequency. There now appeared from the vagina a copious discharge of dark, fetid, grumous water, which satisfied me of the death of the fœtus. Gave a draught of ammonia, ether, and morphia. She slept at intervals till the morning of the 5th, when her exhausted condition determined me to set about completing delivery without further delay ; but prior to deciding upon this step, I consulted with my neighbor Mr. Ayres as to the best mode of accomplishing the object. At this time, Mrs. —— was exhibiting all the symptoms of exhaustion : the pulse at the wrist was 160 ; tongue parched and brown ; skin hot, and of a brick-dust color ; constant delirium of a low rambling kind, interrupted occasionally by lucid intervals, when she implored that decisive steps for the completion of the labour might be adopted. The os tincæ was thoroughly dilated, but the head had not made any considerable descent ; the mischief which was being produced by this long-continued pressure on the soft parts was everywhere to be detected ; the crepitation which I described as existing, on the 4th, in the vaginal mucous membranes, had now resulted in a fissure, an inch and a half long, through the mucous membrane, extending into the cellular tissue. The perinæum was intensely swollen and covered with vesicles, involving the whole margin of the anus ; the bladder had been entirely dependent on the use of the catheter since its first introduction. Feeling satisfied that the fœtus was dead, and the case being one which called for immediate interference, craniotomy suggested itself to me as the only practice applicable ; with such a rigid and inflamed condition of the soft parts, I feel certain that the forceps could not have been successfully employed in the hands of the most accomplished obstetric practitioner. At 11 a. m. I commenced the operation of craniotomy, which occupied an hour. My patient, at intervals, manifested perfect consciousness, at other times was lost to all around her. A disposition to flooding showed itself after delivery, which induced me instantly to introduce my hand into the uterus, and remove the placenta, as I was sensibly alive to the feeble powers of my patient. The fœtus exhibited the first symptoms of decomposition. Ordered a draught, with two drachms of sulphuric ether and forty drops of laudanum in a little camphor mixture. In the evening, the nurse reported that she had slept during short intervals, but was only sensible on being especially roused to reply. Ordered a draught at bed-time, with one grain of morphia and two drachms of sulphuric ether in camphor mixture.

6th, 8a. m.—Had passed a restless night. Pulse 170 ; tongue dry ; delirium constant, of a muttering kind. Ordered a mixture with camphor suspended in mucilage, liquor ammon. acetatis, tinct. hyoscamus and nitras ether.; a dose to be taken every four hours. Morphia, 1 gr. in a draught, to be repeated at night.

7th.—Had occasional sleep during the night, with headache and intolerance of light and sounds. Tongue hard and parched ; skin dry; pulse 160 ; bowels confined. Ol ricini statum ; continue the mixture of yesterday. 10 p. m.—Bowels relieved twice. Night draught, with 1 gr. of morphia repeated.

8th and 9th.—Symptoms continue much the same. I have not been able to produce a lengthened sleep ; continue the night draught.

10th.—Passed a tolerably good night, and appears to be going on favourably ; the pulse, however, of the same peculiar frequency, viz., 160, as at the commencement. 6 p. m.—Visited my patient, and found her suffering from symptoms of an urgent kind ; the temperature of the skin had increased to an excessive degree since the morning ; the slightest noise produced alarm and distress ; and the least ray of light induced pain in the head and general disquiet ; the countenance was crimson red, and anxious in its expression ; delirium almost constant ; bowels violently purged ; lochia suppressed ; and the lacteal secretion had never shown itself in the slightest degree, although the mammary gland was healthy, and fully developed ; her position uneasy, accompanied with great restlessness of manner, indicative of only partial sensibility to her state ; pulse 160. The case, from the commencement, was one which filled me with the greatest anxiety and concern, in consequence of the low state of vitality of my patient, and the exhausted condition of the nervous system ; but at no part of my treatment did I feel greater responsibility than now, believing as I do that a depressing line of practice in these cases, either by bleeding or medicines, if misapplied, is never to be redeemed. Here were a set of symptoms, which have been so ably described as the representatives of inflammation, the treatment of which has also been equally emphatically laid down, namely, bleeding, with depressents ; thus, with the popular ideas of inflammation, and the fondness for active treatment in the public mind, a young practitioner, with equal horrors as myself of the indiscreet use of the lancet, may often have been forced into such a line of treatment to please the multitude, or to protect himself from censure, in the event of an unfavorable issue to the case.

After a little reflection, I decided upon carrying out the tonic and sedative line of treatment, and, with these views, I ordered two pills, containing one and a-half grains of morphia, five grains of camphor, and two of capsicum, to be taken immediately, followed by decoction of bark, with laudanum, aromatic confection, and nitric acid every four hours.

11th, 9 a. m.—Passed a quiet night, getting occasional sleeps of two hour's duration ; bowels still purged, skin moist, pulse 150. Ordered the mixture of bark to be continued, and a draught with one grain of morphia to be taken at night.

12th.—Passed a better night ; bowels only acted on once, skin moist. From this time, Mrs. —— advanced favourably, though slowly, to a state of convalescence.

In addition to the weightier circumstances of this case, there are one or two minor points which strike me as interesting in a practical point of view. The first is, the condition of the pulse at the wrist, which manifested a determined frequency for an unusual length of time. My patient's circulation in health was never more than 80 ; but after her confinement it numbered 160 for three weeks, varying occasionally ten pulsations after the influence of a narcotic, but regaining its frequency as the day advanced. When a substantial diet-scale could with propriety be adopted, the circulation diminished in a very marked manner, but two months elapsed before it came within the range of 100 ; clearly this was a pulse of debility, as proved by the result.

The next point of interest to me is the condition of the mammary gland ; although so highly developed, and my patient, when in health, of a sanguine temperament, yet no trace of any lacteal secretion ever showed itself through her illness ; this I have observed in other cases, and I have also observed that, when this is the case, there has been either an excessive lochial discharge, or flooding, which has rendered the system incapable of the production of the lacteal secretion, or scarcely any appearance of lochia, dependent upon a previous state of inanition of the patient, which has impressed the friends and nurse with the idea of inflammation being in the distance. In this case the same cause which gave rise to all the other symptoms must explain the non-appearance of the lacteal secretion.

The cerebral wanderings and excessive watchfulness all showed the prostrate condition of the powers of the constitution, and the difficulty, in certain idiosyncrasies, to be quieted down, when once deranged, into the natural functions of life. Other cases I could instance of the same tendency, and of great interest, but deem those I have cited sufficient to indicate the value I set upon a liberal and protective treatment.—*Lon. Med. Times, June* 21, 1851.

